# Reference spectrophotometric values for glucose-6-phosphate dehydrogenase activity in two-to six-month-old infants on the Thailand-Myanmar border

**DOI:** 10.12688/wellcomeopenres.18417.1

**Published:** 2022-11-04

**Authors:** Germana Bancone, Day Day Poe, Gornpan Gornsawun, Phyu Phyu Htway, Mary Ellen Gilder, Laypaw Archasuksan, Kesinee Chotivanich, Rose McGready, Francois Nosten

**Affiliations:** 1 Shoklo Malaria Research Unit, Mahidol-Oxford Tropical Medicine Research Unit, Faculty of Tropical Medicine, Mahidol University, Mae Sot, 63110, Thailand; 2 Centre for Tropical Medicine and Global Health, Nuffield Department of Medicine, University of Oxford, Oxford, OX3 7LG, UK; 3 Mahidol-Oxford Tropical Medicine Research Unit (MORU), Faculty of Tropical Medicine, Mahidol University, Bangkok, 10400, Thailand

**Keywords:** G6PD deficiency, Plasmodium vivax, malaria elimination, infants, paediatric G6PD reference values, G6PD testing

## Abstract

**Background:** Glucose-6-phosphate dehydrogenase (G6PD) deficiency represents a barrier to the full deployment of anti-malarial drugs for vivax malaria elimination and of first-line antibiotics. Lack of established reference ranges for G6PD activity in breast-fed infants puts them at risk of drug-induced haemolysis and restricts access to safe treatment of their mothers.

**Methods:** The present work was undertaken to establish age-specific G6PD normal values using the gold standard spectrophotometric assay to support the future clinical use of tafenoquine in lactating women and safer antibiotic treatment in infants.

**Results:** Spectrophotometric results from 78 healthy infants between the ages of 2 and 6 months showed a trend of decreased enzymatic activity with increasing age and provided a reference normal value of 100% activity for infants 2–6 months old of 10.18IU/gHb.

**Conclusions:** Normal reference G6PD activity in 2–6-month-old infants was approximately 140% of that observed in G6PD normal adults from the same population. Age specific G6PD activity thresholds should be used in paediatric populations to avoid drug-induced haemolysis.

## Introduction

Malaria is a leading cause of morbidity and mortality in many developing countries with an estimated 300 to 500 million clinical cases worldwide every year. Tafenoquine is an 8-aminoquinoline antimalarial drug developed by GlaxoSmithKline (GSK) in collaboration with the Medicines for Malaria Venture and 60 Degrees Pharmaceuticals (60°P; ( Chu & Freedman, 2019)), that provides radical cure of *Plasmodium vivax* ( *P. vivax*) by eliminating dormant parasites in the liver and thereby preventing relapses ( Lacerda *et al.*, 2019; Llanos-Cuentas *et al.*, 2019).

The 8-aminoquinoline class of drugs, including primaquine and tafenoquine, is haemolytic in subjects with glucose-6-phosphate dehydrogenase (G6PD) deficiency. G6PD deficiency is the most prevalent human enzymopathy, affecting nearly 400 million people (8% allelic frequency worldwide, ( Howes *et al.*, 2012)) and reaching prevalence over 20% in populations living in malaria endemic countries. Biochemical and genetic studies have identified over 200 genetic variants ( Gómez-Manzo *et al.*, 2016). The consequences of G6PD deficiency in humans are most apparent in red blood cells. Affected individuals are largely asymptomatic and otherwise healthy. However, a precipitating oxidative stress may cause haemolytic anaemia ( Cappellini & Fiorelli, 2008). Common causes of this haemolytic reaction are infections, certain medicines (e.g. anti-malarial compounds such as 8-aminoquinolines and antibiotics), chemicals (e.g. naphthalene in newborns) or foods (e.g. fava beans). G6PD deficiency is also associated with increased risk of moderate and severe neonatal hyperbilirubinaemia by a mechanism not completely understood and only partly explained by haemolysis ( Kaplan & Hammerman, 2002; Luzzatto *et al.*, 2020).

The G6PD gene is located on the X-chromosome. Males with G6PD deficiency are hemizygotes and will only carry deficient red blood cells. Adult male hemizygotes and female homozygotes, with <30% of normal G6PD activity, can usually be detected by the inexpensive fluorescent spot test (FST, ( Beutler & Mitchell, 1968)) or G6PD RDTs ( Ley *et al.*, 2019). However heterozygous females have both a normal and deficient population of red cells (genetic mosaics) resulting in a range of G6PD activity. Females with G6PD activity in the range of 30–70% of normal activity, while susceptible to drug-induced haemolysis ( Chu *et al.*, 2018), are generally miss-classified as normal by FST. Because of increased enzymatic activity at birth ( Doherty *et al.*, 2014), the FST also performs poorly in newborns, missing even fully deficient neonates ( Thielemans *et al.*, 2018). Performance of FST in infant blood is unknown but might be subject to the same shortcomings, especially during the first 6 months of life.

Normal G6PD activity reference values are established by gold standard spectrophotometric assay ( Beutler *et al.*, 1977) and the 30% and 70% thresholds are derived from the median activity obtained in normal males ( Domingo *et al.*, 2013). While standard 14-days primaquine regimen for radical cure of vivax malaria can safely be given to most adults with normal FST results (e.g. ≥ 30% enzymatic activity), safe administration of shorter high-dose primaquine treatment and tafenoquine requires identification of patients with at least 70% of normal G6PD enzymatic activity ( Rueangweerayut *et al.*, 2017). Treatment with other haemolytic drugs, including first-line antibiotics, are generally considered safe in FST-normal subjects but data are inconsistent ( Bancone & Chu, 2021).

There is a paucity of data on G6PD activity levels during the first year of life. Early literature indicates that G6PD activity declines from higher neonatal levels to “adult” levels during this period ( Gross & Hurwitz, 1958; Stave & Pohl, 1960). Travis and colleagues described the changes in G6PD activities in 10 infants tested at 6 time points during the first year of life ( Travis *et al.*, 1980) showing median activities in the first week of life equivalent to 160% of adult values, decreasing to roughly 140% of adult values at 3 months and reaching near-adult values after 6 months of life. A study published in 2020 ( Yang *et al.*, 2020) showed a similar trend in decreasing enzymatic activity during the first 3 months of life in 410 infants aged 7 to 90 days. This data gap has implications for treatment, in particular for malaria, in both infants and their mothers. Lactating women are excluded from radical cure of *P. vivax* because of the theoretical risk of iatrogenic haemolysis in the breastfed infant ( WHO, 2022). Lack of accurate diagnostics for G6PD deficiency in infants and lack of pharmacokinetic data in breast milk prevents appropriate treatment for women during the post-partum period.

G6PD deficiency is highly prevalent in the Karen and Burman populations attending SMRU clinics along the Thailand-Myanmar border ( Bancone *et al.*, 2014; Bancone *et al.*, 2017a), with an estimated prevalence of 9–18% in males; the main mutation found in this population is Mahidol (487G>A) which is associated to a residual enzymatic activity of <30% of normal in hemizygous mutated males. SMRU has recently demonstrated negligible exposure to primaquine in breastfed infants of mothers receiving primaquine ( Gilder *et al.*, 2018); these findings support radical curative treatment of *P. vivax* in breastfeeding women to enhance malaria elimination. Up-coming studies involving treatment of lactating women with tafenoquine will require the assessment of paediatric G6PD normal reference values in order to include breastfed infants <1 year of age. Furthermore, reference values in infants will be helpful to analyse haemolytic effects of other drugs commonly used in this portion of the population.

The current work was undertaken to establish age-specific G6PD normal values in infants using the gold standard spectrophotometric assay to support clinical use of tafenoquine in lactating women and safer antibiotic treatment in infants.

## Methods

### Study design

This was a laboratory study to establish G6PD normal values in infants aged two to six months designed to collect a single blood sample in 80 consecutive healthy male infants attending the Shoklo Malaria Research Unit (SMRU) clinic. The study was conducted SMRU’s Maw Ker Thai clinic situated along the Thailand-Myanmar border in Tak province (Thailand) starting in October 2020. In SMRU clinics free care is provided for migrant populations.

### Participants

Mothers of male infants were approached to enter the study by SMRU clinical staff at standard Expanded Programme of Immunization visits scheduled for 2, 4 and 6 months or study follow-up visits. Staff explained to the mothers of infants the rationale of the study and the blood sampling procedures in their local language, and the mothers were asked to sign a consent form to participate in the research.

Infants were included if they were in the appropriate age range (2–3 months and 4–6 months old) and were healthy (no severe illness, no fever, no diarrhoea, no vomiting). Hospitalization in the previous 3 months, antibiotic use in the last 2 weeks and prematurity (EGA< 37 weeks’ gestation at birth) were further exclusion criteria.

### Ethical approval and consent

The study was approved by Oxford Tropical Research Ethics Committee, UK (OxTREC 543-19), the Mahidol University Faculty of Tropical Medicine Ethics Committee, Thailand (TMEC 19-047, MUTM 2019-079-01) and the Tak Province Border Community Ethics Advisory Board (TCAB201911). Written informed consent was obtained from literate mothers; a thumbprint was obtained in the presence of a literate witness for illiterate mothers.

### Data collection

A single 100µl (2–3 drops of blood) capillary blood sample was collected by heel-prick into an EDTA-treated Safe-T-fill ^®^ capillary tube. Samples were stored at 4°C at the clinics until transported refrigerated (within 8 hours) to the central haematology lab of SMRU. At the central haematology laboratory, blood was analysed by gold standard spectrophotometric assay and complete blood count (CBC) or Hemocue 301 ^+^ system within 24 hours from collection. The Pointe Scientific spectrophotometric assay was used (assay kit # G7583-180, Lysis Buffer # G7583-LysSB). Kinetic determination of G6PD activity at 340 nm was performed using a SHIMAZU UV-1800 spectrophotometer with temperature-controlled cuvette compartment (30°C). Samples were analysed in duplicate and mean activity was expressed in IU/gHb using the Hb concentration obtained by CBC or Hemocue 301+. The final result was calculated using manufacturer’s Temperature Control Factor of 1.37. Two controls (Normal, Intermediate or Deficient; Analytic Control Systems, Inc. USA) were analysed at every run and results compared to expected ranges provided by manufacturer.

CBC was performed using a CeltacF MEK-8222K haematology analyser (Nihon Kohden, Japan). Three-level quality controls were run every day and device maintenance and calibration were performed regularly. Hemocue 301+ was run according to manufacturer’s instructions.

### Sample size and statistical analyses

The proposed sample size was calculated from the following formula to estimate the mean of a population with a 95% CI of [mean± δ]:

N= (z ^2^σ ^2^)/δ ^2^

Where z=1.96 and σ is the expected standard deviation.

We used a σ=2 and δ=0.75, so our minimum sample was 28. To account for exclusions from analysis of G6PD deficient subjects (around 15–18% of the male population) and a 10% invalid results, a total of 40 subjects was planned to ensure a total of 30 evaluable subjects.

Two age groups were evaluated, one group included infants aged from 2 months + 0 days to 3 months + 14 days (herein called “2–3 months old group”) and a second group included infants aged from 4 months + 0 days to 6 months + 14 days (i.e. “4–6 month old groups”).

Anaemia was defined as haemoglobin concentration <10.0 g/dL.

Male median equivalent to reference 100% activity was calculated as the median of activity in G6PD normal males. Medians were compared by Mood’s test and Mann-Whitney test.

Categorization of enzymatic activities in 5 classes (< 6.0 IU/gHb, 6.0–7.9IU/gHb, 8.0–9.9IU/gHb, 10.0–11.9IU/gHb and ≥12IU/gHb) was chosen arbitrarily to cover the observed range of activities in both newborns and adult specimens from G6PD normal subjects.

Comparison of data from the current study with data on male infants in published literature was performed on studies that used different laboratory methodologies to assess G6PD enzymatic activity ( Gross & Hurwitz, 1958; Konrad *et al.*, 1972; Stave & Pohl, 1960; Tang *et al.*, 1992; Travis *et al.*, 1980; Yang *et al.*, 2020). In order to compare results, percentage enzymatic activity was obtained by normalising newborns and infant data from each study to their reported normal value in adults.

## Results

**Figure 1.**
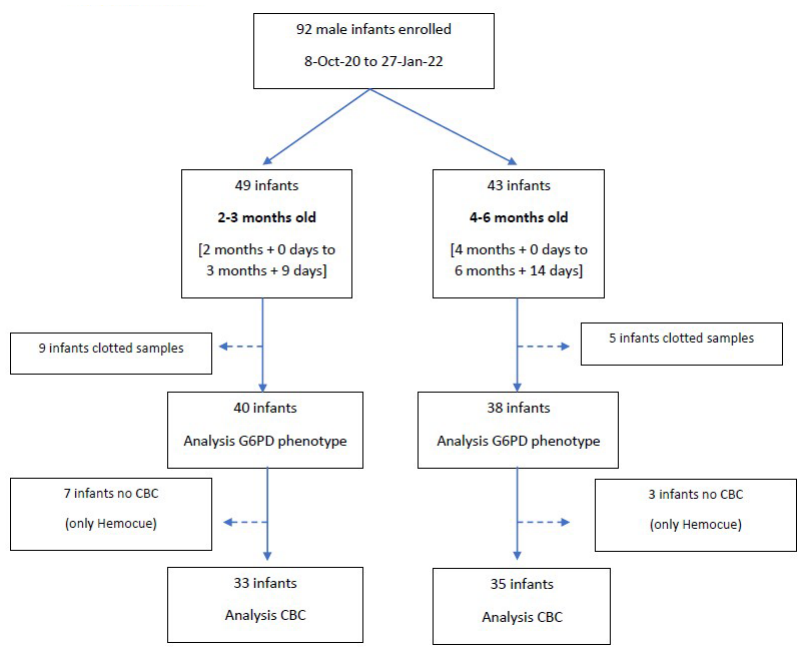
Study flow.

Rate of enrolment was heavily influenced by the COVID-19 ongoing pandemic and the travel restrictions imposed during the years 2020 and 2021. From 8 October 2020 to 24 June 2021 and from 16 September to 27 January 2022, a total of 92 male infants (49 in the 2–3months old group and 43 in the 4–6 months old group) were enrolled in the study; blood samples from 9 infants in the 2–3 months old group and 5 infants in the 4–6 months old group were rejected because they were clotted. Analyses were performed on specimens from 78 infants (40 in 2–3 months old group and 38 in 4–6 months old group, Figure 1). Median (min-max) age was 65.0 (60–101) days in the 2–3 months old group and 130 (118–199) days in the 4–5 months old group. The full dataset can be found under *Underlying data* ( Bancone *et al.*, 2022a, “GNB study database”).

### Haematological characteristics

White blood cells and platelets counts in infants of different age were similar; red blood cell (RBC) count was significantly higher in older infants and mean corpuscular volume (MCV) and MCH (mean corpuscular haemoglobin) significantly lower in older infants ( Table 1) reflecting different distribution of RBC ages in the two groups.

**Table 1.** Mean (SD) blood indices by age group.

Age	N ^a^	WBC (10 ^3^/ uL)	RBC (10 ^6^/ uL)	HGB (g/dL)	HCT (%)	MCV (fL)	MCH (pg)	MCHC (g/dL)	RDW (%)	PLT (10 ^3^/ uL)
2–3 months	33	7.6 (1.9)	4.2 (0.5)	10.8 (1.1) [40]	37.4 (4.0)	89.5 (7.0)	25.5 (2.3)	28.4 (1.3)	15.4 (1.5)	270 (107)
4–6 months	35	7.5 (2.1)	4.7 (0.4)	10.4 (1.0) [38]	36.5 (3.2)	78.1 (6.8)	22.2 (2.3)	28.3 (1.4)	14.8 (1.8)	256 (72)
*p*		0.790	<0.001	0.096	0.313	<0.001	<0.001	0.728	0.138	0.507

*^a^ A total of 10 infants (7 in the 2–3 months group and 3 in the 4–6 months group) did not have a CBC available; Hb was assessed by Hemocue 301+ instead.*

Anaemia defined by haemoglobin levels <10.0g/dL was common in both groups, 30.0% (12/40) in younger infants and 39.5% (15/38) in older infants. Overall, 50% (4/8) of G6PD deficient infants were anaemic compared with 32.9% (23/70) of the G6PD normal infants.

### G6PD activity

**Figure 2.**
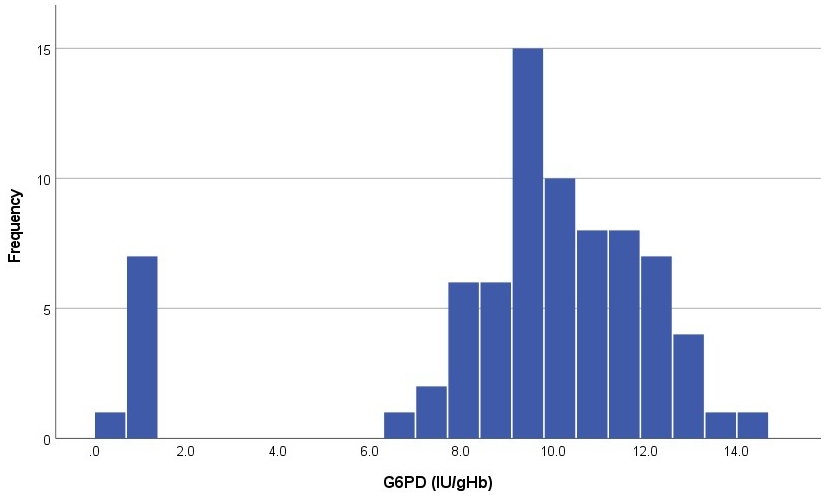
Distribution of G6PD activity (IU/gHb) in the analysed samples.

The distribution of G6PD activity showed a clear bimodal shape with 10.3% (8/78) infants in the left peak (G6PD deficient) with an activity less than 1.3IU/gHb ( Figure 2).

Male median (min-max) enzymatic G6PD activity was 10.53 (7.51-14.97) IU/gHb in 37 G6PD normal infants of 2–3 months old group, and 9.74 (6.98-12.95) IU/gHb in 33 G6PD normal infants of 4–6 months old group (Exact, 2-sided: Mood’s test P=0.150; Mann-Whitney test P=0.259) ( Table 2).

**Table 2.** Median (min-max) enzymatic activity in G6PD deficient and normal infants in the two age groups.

Age	G6PD phenotype	N	Median	Minimum	Maximum
2–3 months	Deficient	3	0.82	0.68	1.15
	Normal	37	10.53	7.51	14.97
4–6 months	Deficient	5	1.01	0.89	1.28
	Normal	33	9.74	6.98	12.95
All	Deficient	8	0.96	0.68	1.28
	Normal	70	10.18	6.98	14.97

The established male median from the whole sample allowed definition of the 30% and 70% activity threshold for infants 2–6 months of age corresponding to 3.05IU/gHb and 7.13 IU/gHb.

**Figure 3.**
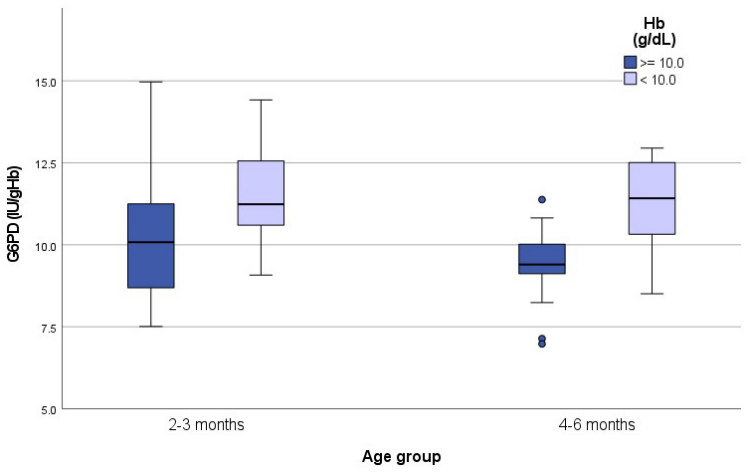
G6PD activity (IU/gHb) in anaemic and non-anaemic infants according to age groups. Median G6PD activity in non-anaemic infants was 10.1IU/gHb in group 1 and 9.4IU/gHb in group 2. In anaemic infants activity was11.2IU/gHb in group 1 and 11.4IU/gHb in group 2.

G6PD activity is normalized using haemoglobin concentrations therefore results in anaemic subjects can be falsely elevated ( Bancone *et al.*, 2017b) as plotted in Figure 3.

### G6PD activity in neonates, infants and adults

**Figure 4.**
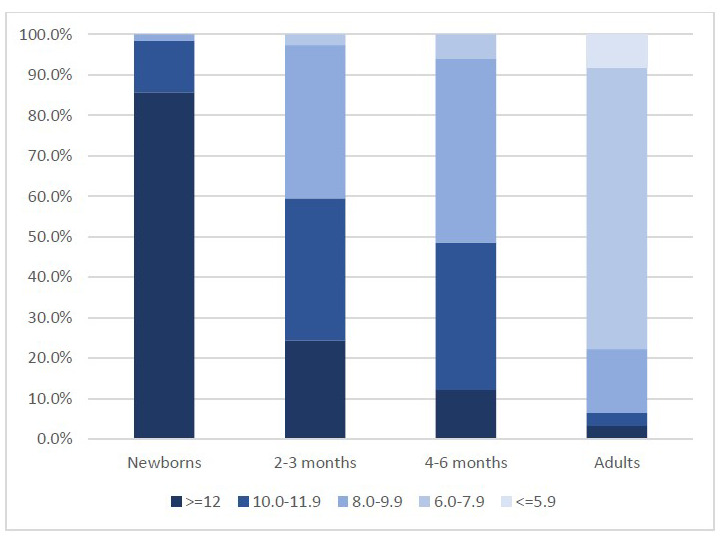
Distribution of G6PD activity (IU/gHb) categories according to age groups. Data in newborns were collected during a recent study in SMRU clinics involving 125 G6PD normal male neonates; data in adults were collected during a recent study in SMRU clinics involving 95 G6PD normal healthy males.

In G6PD normal infants, G6PD activity classified in 5 categories (< 6.0 IU/gHb, 6.0-7.9IU/gHb, 8.0-9.9IU/gHb, 10.0-11.9IU/gHb and ≥12IU/gHb) showed a trend of different distribution by age group as compared to samples previously collected in the same setting among newborns ( Bancone *et al.*, 2022b) and healthy adults (unpublished data) as shown in Figure 4.

**Figure 5.**
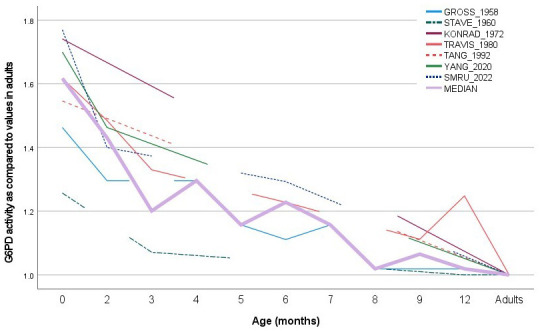
G6PD enzymatic activity at different ages: comparison of current data with published data. G6PD enzymatic activity was normalized by levels assessed at 1 year of age or in adults. The continuous pink line represents the median calculated from all published and current data ( Table 3).

Data from the current study were also compared to published literature that reported reference G6PD enzymatic activity assessed in term newborns, infants up to one year of life and adults (where available) within the same study ( Figure 5).

**Table 3.** G6PD enzymatic activity at different ages: comparison of current data with published data.

Age (months)	birth	2	3	4	5	6	7	8	9	12	Adults	Total N
Gross & Hurwitz, 1958	1.463 (N=24)	1.296 (N=4)		1.296 (N=8)	1.157 (N=2)	1.111 (N=3)	1.157 (N=2)	1.019 (N=5)	1.019 (N=3)	1.019 (N=4)	1.000 (N=17)	72
Stave & Pohl, 1960	1.257 (N=11)		1.071 (N=8)							1.000 (N=9)	1.000 (N=10)	38
Konrad *et al.*, 1972	1.741 (=22)										1.000 (N=50)	72
Travis *et al.*, 1980	1.617 (N=10)	1.485 (N=10)	1.330 (N=10)			1.228 (N=10)			1.112 (N=10)	1.248 (N=10)	1.000 (N=10)	10 *
Tang *et al.*, 1992	1.546 (N=NR)										1.000 (N=NR)	NR
Yang *et al.*, 2020	1.700 (N=70)	1.463 (N=148)									1.000 (N=120)	338
SMRU data	1.770 (N=125)	1.400 (N=37)				1.293 (N=33)					1.000 (N=95)	290
Unweighted median	1.617	1.431	1.201	1.296	1.157	1.228	1.157	1.019	1.065	1.019	1	

*The same 10 infants were followed-up over time. NR= non reported

## Discussion

This was the first study to assess quantitative G6PD activity in infants outside the newborn period in this population, and represented, to our knowledge, the largest published dataset of G6PD activity in 4–6-month-old infants. When compared to median G6PD activity at birth assessed in a larger cohort in the same population (13.3IU/gHb) and reference male median in adults (7.5IU/gHb), the male median observed in 2–3-month-old infants (10.5IU/gHb) and in 4–6-month-old infants (9.7IU/gHb) confirmed a trend of gradual decline of enzymatic activity during infancy. Enzymatic activity at birth was over 170% of the adult levels and declined to 140% of adult levels by 6 months of age. This trajectory was comparable with previously collected data and consistent with a higher proportion of younger RBCs in newborns and/or shorter lifespan in erythrocytes from younger infants ( Kuruvilla *et al.*, 2017).

Importantly, when observing increased levels of G6PD activity, thresholds for definition of “G6PD deficiency” increased proportionally; should adult thresholds be applied to infants, deficient and intermediate infants would be inappropriately classified as normal. Misclassification of deficient individuals as normal could put them at risk for drug-induced hemolysis if the G6PD normal misdiagnosis follows them through childhood and adulthood. It is unknown whether higher enzymatic activity observed in infancy would provide some protection from oxidative haemolysis in this phase of life.

Though much progress has occurred in the past decade towards *P. falciparum* elimination in the populations seeking care at SMRU’s clinics, *P. vivax* has resisted elimination efforts due to the persistence of hypnozoites and operational barriers to broad implementation of radical cure. Following over one year of political unrest, Myanmar and the bordering countries have already seen increasing clinical cases of *P. vivax*, as similarly observed during armed conflicts in Africa ( Sedda *et al.*, 2015). Radical cure for vivax malaria using single-dose tafenoquine has an obvious advantage in treatment adherence compared to 14-days but also 7-day primaquine courses; this becomes even more relevant for mobile populations living in areas with decreased security and poor health systems. A sizeable proportion of the population which lacks access to radical vivax treatment is represented by women of reproductive age ( Brummaier *et al.*, 2020), especially when they experience multiple pregnancies in close succession; a safe radical cure should be provided during lactation ( Watson *et al.*, 2018) and the result of this study will support clinical trials using tafenoquine in breastfeeding mothers.

This work established reference normal values for G6DP activity in infants of 2 to 6 months of age, after which G6PD activity was expected to approach adult levels. However, median G6PD activity in 6-month-old infants from this cohort appears to still be well above the adult median (10.2IU/gHb corresponding to 140% of adult levels), suggesting that more data in late infancy and anaemic infants would be valuable.

G6PD point-of-care quantitative tests have been recently evaluated for use in newborns and their evaluation in infants would provide additional indication for use in non-adult patients. Use of the same G6PD point-of-care device across different settings ( Ley *et al.*, 2022) would bypass the need for locally established paediatric reference ranges for G6PD activity which presents an added barrier to diagnosis in children.

## Conclusions

G6PD reference ranges in infants 2 to 6 months of age were established locally to support expansion of radical curative treatments of vivax malaria in lactating mothers, starting with clinical trials to quantify the amount of drug present in breast milk. Reference ranges will also be useful to improve treatment with potentially haemolytic drugs (e.g. antibiotics) in pediatric patients. More work will need to be done to establish reference ranges for up to 1 year of life and to assess use of quantitative point-of-care G6PD tests in infants.

## Data Availability

Zenodo: GNB study database. https://doi.org/10.5281/zenodo.7181827. ( Bancone *et al.*, 2022a) This project contains the following underlying data: Dataset.xlsx (De-identified raw data) Data are available under the terms of the Creative Commons Attribution 4.0 International license (CC-BY 4.0).
