# Supplementary material for: Reference spectrophotometric values for glucose-6-phosphate dehydrogenase activity in two-to six-month-old infants on the Thailand-Myanmar border
Source: Wellcome Open Res. 2024 Jan 9;7:273. Originally published 2022 Nov 4. [Version 2] doi: 10.12688/wellcomeopenres.18417.2 (PMC10884598; doi:10.12688/wellcomeopenres.18417.2)
Supplement: Peer review history

# Reviewer report: Reviewer response for version 2

**DOI:** 10.21956/wellcomeopenres.23073.r72442

**Published:** 2024-02-22

**Authors:** Collins Ouma

**Affiliations:** 1 University of New Mexico-Kenyan Global Health Programs Laboratories, New Mexico, Kenya

No further comment

Is the work clearly and accurately presented and does it cite the current literature?

Partly

If applicable, is the statistical analysis and its interpretation appropriate?

No

Are all the source data underlying the results available to ensure full reproducibility?

Partly

Is the study design appropriate and is the work technically sound?

Yes

Are the conclusions drawn adequately supported by the results?

Partly

Are sufficient details of methods and analysis provided to allow replication by others?

Partly

Reviewer Expertise:

Biomedical sciences, infectious and non-infectious diseases, genetics

I confirm that I have read this submission and believe that I have an appropriate level of expertise to confirm that it is of an acceptable scientific standard.

# Reviewer report: Reviewer response for version 1

**DOI:** 10.21956/wellcomeopenres.20422.r63167

**Published:** 2023-09-20

**Authors:** Vinod K Bhutani

**Affiliations:** 1 Stanford University School of Medicine, Stanford, CA, USA

**General Comments** Maternal-child-health perspectives of living safely with inherited Glucose-6-Phosphate Dehydrogenase (G6PD) deficiency remains a poorly investigated area in both medical and public health research disciplines. Bancone et al provide a valuable contribution to address the lack of established reference ranges for G6PD activity in breast-fed infants who are at risk of drug-induced and other environmental induced hemolysis. This condition not only has direct adverse impact on babies but also restricts access to safe intervention options of their mothers.

 The authors could have highlighted a wider impact of their data such that future research could be directed at: a) newborn babies who are immediately initiated on breast feeding; b) on the safety among mothers who most likely have varying degree of enzyme deficiency because of the complex inheritance of this X-linked genetic condition; c) collection of potential adverse outcome data among newborns and infants who are inadvertently exposed to unsafe anti-malarial drugs; and, d) importantly, expand the study age range of the cohort to study both neonatal and infancy phases and trajectory of G6PD enzyme activity concurrent with the natural decline in hemoglobin concentrations.

 Thus, this body of work is not just limited to the public health/infection disease community caring for malarial prophylaxis only. The authors are commended to help define the assessment of pediatric G6PD normal reference values in order to include breastfed infants <1 year of age. By this token, the report should extend **from age 0 to 364 days**. Since only male infants were studied, exclusion by gender is problematic. At this stage, limiting the cohort to term and late-preterm neonates is appropriate. However, future opportunities to study safety of breastfeeding premature infants should be sought. I do understand the malaria perspective but the MCH perspective in an endemic area is of much wider. significance This is a good start. **I agree **with most of the comments by Collins Ouma.

**Specific Comments:** I suggest additional **considerations **inclusive of clarifications:

1. **Reference by age: **The present work has been undertaken to establish age-specific-G6PD enzyme (specify by days, weeks or months) normal values using the gold standard spectrophotometric assay to support the future clinical applications to identify the enzyme deficiency status in lactating women and their infants. Do you have data for the mothers?
2. **Deficiency threshold: **Spectrophotometric results from 78 healthy infants between the ages of 2 and 6 months showed a trend of decreased enzymatic activity with increasing age (in days, weeks or months). These data provide a “reference” normal (mean, median?) value of 100% activity for infants 2-6 months (as a single cohort) old of 10.18 IU/gHb (is this the mean or median, add SD, range?). Is there a statistical basis to offer a specific ‘deficiency threshold? Could the authors suggest a statistical out-of-range value to guide a “deficiency” threshold? How is gender of the cohort handled?
3. **Timeliness: **Can the authors highlight the total turn-around time (collection of blood to clinical action upon results) for enzyme assay results since a timed interval is critical to support the least interruption of lactational care?
4. **Gender considerations:** Normal reference G6PD activity in 2–6-month-old male infants was approximately 140% of that observed in G6PD normal adults (just males? What about non-pregnant females, pregnant mothers, or lactating mothers from the same population?). Age specific G6PD activity thresholds should be used in both male and female pediatric populations so that the goal would be to achieve healthy outcomes and prevent environmental, diet and chemical induced hemolysis in the entire population.
5. **Study Cohort: **The rationale for sub-dividing the cohort is unclear and needs explanation to merit a statistical comparison (Table 2). Perhaps, the data could be presented of the two cohorts: deficient and “normal” [as per by the bi-modal differentiation {Figure 2}] in relation to post-natal age (in days) in format of mean/median and confidence interval or range.
6. **Confounding factors: **Since the authors have focused on a specific target population, is the emphasis related to ethnicity, ancestral heritage or related endemicity, geography or socio-economic factors related to literacy as well integrity of self-care health practices?

Is the work clearly and accurately presented and does it cite the current literature?

Partly

If applicable, is the statistical analysis and its interpretation appropriate?

Partly

Are all the source data underlying the results available to ensure full reproducibility?

Yes

Is the study design appropriate and is the work technically sound?

Yes

Are the conclusions drawn adequately supported by the results?

Yes

Are sufficient details of methods and analysis provided to allow replication by others?

Yes

Reviewer Expertise:

Paediatrics and Neonatology

I confirm that I have read this submission and believe that I have an appropriate level of expertise to confirm that it is of an acceptable scientific standard.

# Response

**Published:** 2024-01-04

**Authors:** Germana Bancone

**Affiliations:** Shoklo Malaria Research Unit, Thailand

Thanks for reviewing our manuscript, please find below our answers to the comments: *“The authors could have highlighted a wider impact of their data such that future research could be directed at: a) newborn babies who are immediately initiated on breast feeding; b) on the safety among mothers who most likely have varying degree of enzyme deficiency because of the complex inheritance of this X-linked genetic condition; c) collection of potential adverse outcome data among newborns and infants who are inadvertently exposed to unsafe anti-malarial drugs; and, d) importantly, expand the study age range of the cohort to study both neonatal and infancy phases and trajectory of G6PD enzyme activity concurrent with the natural decline in hemoglobin concentrations.”* We sincerely appreciate the suggestions and have added some points to the discussion; nevertheless, we do not want to stretch the impact of this small study beyond its significance. We would also like to direct the reviewer to a manuscript published by our group in which we evaluate a point-of-care G6PD test for use in neonates which is likely to improve neonatal clinical care for jaundice in LMIC ( Technical evaluation and usability of a quantitative G6PD POC test in cord blood: a mixed-methods study in a low-resource setting - PubMed (nih.gov)) which addresses in part some of the issues raised by the reviewer. 

*“By this token, the report should extend **from age 0 to 364 days**.”* This would be very interesting and would provide useful data but, unfortunately, we do not have data available up to 12 months of life. As reported to the other reviewer, a) our main focus, stated throughout the manuscript, was to obtain reference ranges of G6PD activity in the specific population of infants up to 6 months of age;  b) we did not know before obtaining the data that the G6PD activity in 6-months old infants had not reached levels comparable to adults; c) the clinical setting in which we work does not have a pediatric clinic and our capacity to include healthy children older than 6 months is very limited. We have clarified this last point in the methods section. *“Since only male infants were studied, exclusion by gender is problematic”* This is indeed a very common approach when assessing normal reference values of G6PD enzymatic activity; due to the natural bimodal distribution in G6PD activitiy in a population of random males, it is easier to select G6PD normal males without genotyping and to establish normal reference value. This allows to keep a small sample size and obtain data that can be then used for the whole population (males and females). Please see G6PD testing in support of treatment and elimination of malaria: recommendations for evaluation of G6PD tests - PubMed (nih.gov) *“At this stage, limiting the cohort to term and late-preterm neonates is appropriate. However, future opportunities to study safety of breastfeeding premature infants should be sought. I do understand the malaria perspective but the MCH perspective in an endemic area is of much wider significance This is a good start. **”*** We fully agree with the reviewer and hope to be able in future to investigate more in this direction. 

***Reference by age:*** *The present work has been undertaken to establish age-specific-G6PD enzyme (specify by days, weeks or months) normal values using the gold standard spectrophotometric assay to support the future clinical applications to identify the enzyme deficiency status in lactating women and their infants. Do you have data for the mothers?* We already have reference G6PD values established in the adult population and will use them for safe treatment of lactating mothers. This study was undertaken to establish normal values in infants only. ***Deficiency threshold:*** *Spectrophotometric results from 78 healthy infants between the ages of 2 and 6 months showed a trend of decreased enzymatic activity with increasing age (in days, weeks or months). These data provide a “reference” normal (mean, median?) value of 100% activity for infants 2-6 months (as a single cohort) old of 10.18 IU/gHb (is this the mean or median, add SD, range?). Is there a statistical basis to offer a specific ‘deficiency threshold? Could the authors suggest a statistical out-of-range value to guide a “deficiency” threshold? How is gender of the cohort handled?*

   The 10.18IU/gHb is the median value among normal males and represent the 100% normal reference value G6PD testing in support of treatment and elimination of malaria: recommendations for evaluation of G6PD tests - PubMed (nih.gov). The deficiency threshold of 30% reported in the manuscript follow WHO recommendation for adults of both sex Testing for G6PD deficiency for safe use of primaquine in radical cure of P. vivax and P. ovale (who.int) and what we also used for neonates Technical evaluation and usability of a quantitative G6PD POC test in cord blood: a mixed-methods study in a low-resource setting - PubMed (nih.gov); the 70% threshold is used for safe treatment using Tafenoquine ( Hemolytic Potential of Tafenoquine in Female Volunteers Heterozygous for Glucose-6-Phosphate Dehydrogenase (G6PD) Deficiency ( G6PD Mahidol Variant) versus G6PD-Normal Volunteers - PubMed (nih.gov) ***Timeliness:*** *Can the authors highlight the total turn-around time (collection of blood to clinical action upon results) for enzyme assay results since a timed interval is critical to support the least interruption of lactational care?* In the planned clinical studies, we will be testing G6PD by in-house spectrophotometry with results available within 24 hours.  Primaquine and tafenoquine treatment can be delayed for 1-2 days without adverse clinical effects so this is acceptable for the purpose of the clinical studies. However, we agree with the reviewer’s implication that a rapid test (such as a G6PD biosensor) would be useful in future when treatment of lactating women might become common practice and quantitative testing for both mother and infant will be needed at the point-of-care.  Evaluation of G6PD Biosensor tests in infants will be needed. 

***Gender considerations:*** *Normal reference G6PD activity in 2–6-month-old male infants was approximately 140% of that observed in G6PD normal adults (just males? What about non-pregnant females, pregnant mothers, or lactating mothers from the same population?). Age specific G6PD activity thresholds should be used in both male and female pediatric populations so that the goal would be to achieve healthy outcomes and prevent environmental, diet and chemical induced hemolysis in the entire population.* Yes, the normal threshold is intended to be used by the whole pediatric population, males and females. We have clarified this. The comparison was done with the reference adult levels used for the whole adult population. 

***Study Cohort:*** *The rationale for sub-dividing the cohort is unclear and needs explanation to merit a statistical comparison (Table 2). Perhaps, the data could be presented of the two cohorts: deficient and “normal” [as per by the bi-modal differentiation {Figure 2}] in relation to post-natal age (in days) in format of mean/median and confidence interval or range.* The rationale for sub-dividing the cohort by age came from an effort to maximize participation of healthy infants in the study while attending EPI (this has been clarified in the methods) and the lack of knowledge on what G6PD activity level we could expect at the different ages. We believe Table 2 is already presenting these data.

***Confounding factors:*** *Since the authors have focused on a specific target population, is the emphasis related to ethnicity, ancestral heritage or related endemicity, geography or socio-economic factors related to literacy as well integrity of self-care health practices?* The target population represents a collection of communities with high prevalence of G6PD deficiency that has been impacted by malaria for generations. SMRU has been working for over 3 decades with this population, providing free health care and conducting research that addresses the clinical needs of the local population. In this regard, evaluation of G6PD tests and establishment of relevant normal reference has become an important effort to improve local health by providing safe radical cure for vivax malaria and by supporting neonatal care for jaundice. We confirm, however, that the data apply to this specific population and pediatric reference laboratory ranges might need to be established in different settings.

# Reviewer report: Reviewer response for version 1

**DOI:** 10.21956/wellcomeopenres.20422.r63174

**Published:** 2023-08-18

**Authors:** Collins Ouma

**Affiliations:** 1 University of New Mexico-Kenyan Global Health Programs Laboratories, New Mexico, Kenya

Bancone et al presents findings on a study undertaken to establish age-specific G6PD normal values using the gold standard spectrophotometric assay to support the future clinical use of tafenoquine in lactating women and safer antibiotic treatment in infants. The manuscript can benefit from the following improvements:

1. The Abstract presents data that are not statistically analysed. Statistics that lead to the current conclusions would be critical on the abstract.
2. As much as the study is carried out on children in Thailand, the study site is not mentioned on the abstract. It takes the reader to get back on the title and possibly read through Methods to figure out where the study was performed.
3. Introduction: Authors state that there is paucity on data on G6PD activity levels during the first year of life.  This in my understanding is within the first 12 months of life.  The rationale for narrowing down on children aged between 2-6 months need to be provided. If not provided adequately, I would suggest that data be presented for children within the first 12 months.
4. Recruitment: what happened to children who are HIV-positive but have no stated conditions for exclusions? 
5. The definition of anaemia provided as haemoglobin concentration <10.0g/dL is not referenced. Is this according to WHO definition or regional definition?
6. Categorisation of enzymatic activities is stated to be arbitrary. I think this is not the first time such a study is being done. I would suggest a use of a more standard categorisation.
7. No statistical analyses approaches provided in the methods, not even the level of assessing statistical significance.
8. Results, Discussion and Conclusions: if my comments above are not addressed, the conclusions may sound spurious and as such need to be re-looked.

Is the work clearly and accurately presented and does it cite the current literature?

Partly

If applicable, is the statistical analysis and its interpretation appropriate?

No

Are all the source data underlying the results available to ensure full reproducibility?

Partly

Is the study design appropriate and is the work technically sound?

Yes

Are the conclusions drawn adequately supported by the results?

Partly

Are sufficient details of methods and analysis provided to allow replication by others?

Partly

Reviewer Expertise:

Biomedical sciences, infectious and non-infectious diseases, genetics

I confirm that I have read this submission and believe that I have an appropriate level of expertise to confirm that it is of an acceptable scientific standard, however I have significant reservations, as outlined above.

# Response

**Published:** 2024-01-04

**Authors:** Germana Bancone

**Affiliations:** Shoklo Malaria Research Unit, Thailand

Thanks for reviewing our manuscript, please find below our point-to-point answers to the comments.

1. *The Abstract presents data that are not statistically analysed. Statistics that lead to the current conclusions would be critical on the abstract.*
  We have added this into the abstract.
2. *As much as the study is carried out on children in Thailand, the study site is not mentioned on the abstract. It takes the reader to get back on the title and possibly read through Methods to figure out where the study was performed.*

We have added the location in the abstract.

1. *Introduction: Authors state that there is paucity on data on G6PD activity levels during the first year of life.  This in my understanding is within the first 12 months of life.  The rationale for narrowing down on children aged between 2-6 months need to be provided. If not provided adequately, I would suggest that data be presented for children within the first 12 months.*

It would be very interesting to extend analysis to older children but, unfortunately, we do not have data available up to 12 months. This is explained by the following reasons: a) our main focus, stated throughout the manuscript, was to obtain reference ranges of G6PD activity in the specific population of infants up to 6 months of age;  b) we did not know before obtaining the data that the G6PD activity in 6-months old infants had not reached levels comparable to adults; c) the clinical setting in which we work does not have a pediatric clinic and our capacity to include healthy children older than 6 months is very limited. We have clarified this last point in the methods section.

1. *Recruitment: what happened to children who are HIV-positive but have no stated conditions for exclusions? *
  HIV test was not performed since it is not expected to have an impact on G6PD activity. Furthermore, HIV prevalence in this population is extremely low (< 1% Audit of antenatal screening for syphilis and HIV in migrant and refugee women on the Thai-Myanmar border: a descriptive study - PubMed (nih.gov)).
2. *The definition of anaemia provided as haemoglobin concentration <10.0g/dL is not referenced. Is this according to WHO definition or regional definition?*
  This is WHO definition; we have added the reference in the methods.
3. *Categorisation of enzymatic activities is stated to be arbitrary. I think this is not the first time such a study is being done. I would suggest a use of a more standard categorisation.*
  Categorization in this context had to be arbitrary because we presented data from neonates, infants and adults who have very different normal G6PD activities; a standard categorization used in adults would not be suitable for neonates and vice-versa. It is worth noting that this categorization is only used for the purpose of graphical representation and it is not analysed statistically.
4. *No statistical analyses approaches provided in the methods, not even the level of assessing statistical significance.*
  Statistics for comparison of medians were already reported. We have added that the significance was assessed at 5% level.
5. *Results, Discussion and Conclusions: if my comments above are not addressed, the conclusions may sound spurious and as such need to be re-looked *

We hope we have addressed comments in a satisfactory way and that the conclusions are fully sustained when considering this additional information.
